# High Performance of Ionic-Liquid-Based Materials to Remove Insecticides

**DOI:** 10.3390/ijms23062989

**Published:** 2022-03-10

**Authors:** Rafael Francisco, Catarina Almeida, Ana C. A. Sousa, Márcia C. Neves, Mara G. Freire

**Affiliations:** 1 CICECO-Aveiro Institute of Materials, Department of Chemistry, University of Aveiro, 3810-193 Aveiro, Portugal; rafaelfrancisco@ua.pt (R.F.); ac.almeida@ua.pt (C.A.); mcneves@ua.pt (M.C.N.); 2 Department of Biology, School of Science and Technology, University of Évora, 7006-554 Évora, Portugal; acsousa@uevora.pt; 3 Comprehensive Health Research Centre (CHRC), University of Évora, 7000-671 Évora, Portugal

**Keywords:** neonicotinoids, imidacloprid, acetamiprid, thiacloprid, thiamethoxam, adsorption, removal, supported ionic liquids

## Abstract

Neonicotinoids are systemic insecticides commonly used for pest control in agriculture and veterinary applications. Due to their widespread use, neonicotinoid insecticides (neonics) are found in different environmental compartments, including water, soils, and biota, in which their high toxicity towards non-target organisms is a matter of great concern. Given their widespread use and high toxicity, the development of strategies to remove neonics, while avoiding further environmental contamination is of high priority. In this work, ionic-liquid-based materials, comprising silica modified with tetraalkylammonium cations and the chloride anion, were explored as alternative adsorbent materials to remove four neonics insecticides, namely imidacloprid, acetamiprid, thiacloprid, and thiamethoxam, from aqueous media. These materials or supported ionic liquids (SILs) were first synthesized and chemically characterized and further applied in adsorption studies. It was found that the equilibrium concentration of the adsorbate in the solid phase decreases with the decrease in the SIL cation alkyl chain length, reinforcing the relevance of hydrophobic interactions between ionic liquids (ILs) and insecticides. The best-identified SIL for the adsorption of the studied insecticides corresponds to silica modified with propyltrioctylammonium chloride ([Si][N_3888_]Cl). The saturation of SILs was reached in 5 min or less, showing their fast adsorption rate towards all insecticides, in contrast with activated carbon (benchmark) that requires 40 to 60 min. The best fitting of the experimental kinetic data was achieved with the Pseudo Second-Order model, meaning that the adsorption process is controlled at the solid-liquid interface. On the other hand, the best fitting of the experimental isotherm data is given by the Freundlich isotherm model, revealing that multiple layers of insecticides onto the SIL surface may occur. The continuous removal efficiency of the best SIL ([Si][N_3888_]Cl) by solid-phase extraction was finally appraised, with the maximum adsorption capacity decreasing in the following sequence: imidacloprid > thiacloprid > thiamethoxam > acetamiprid. Based on real reported values, under ideal conditions, 1 g of [Si][N_3888_]Cl is able to treat at least 10^6^ m^3^ of wastewater and water from wetland contaminated with the studied neonics. In summary, the enhanced adsorption capacity of SILs for a broad diversity of neonics was demonstrated, reinforcing the usefulness of these materials for their removal from aqueous matrices and thus contributing to preventing their introduction into the ecosystems and reducing their detrimental effects in the environment and human health.

## 1. Introduction

Neonicotinoid insecticides (neonics) are one of the most used classes of insecticides worldwide for pest control in residential and agricultural fields [1,2]. They appeared in the 1990s, reaching a market share of more than 25% of the global insecticides sales in 2014, with an annual global sale of more than $3.5 billion [2,3,4].

All neonics present a similar structure to nicotine, the molecule in which they were first inspired. Imidacloprid, acetamiprid, and thiacloprid exhibit the same cloropyridine moiety as nicotine, whereas thiamethoxam comprises a clorothiazole group [2]. Neonics can be classified into three main groups, namely N-nitroguanidines that include, for example, imidacloprid and thiamethoxam, nitromethylenes, and N-cyanoamidines, which comprises, for instance, acetamiprid [3].

Due to their broad-spectrum of insecticidal activity, the versatility of application modes (e.g., in spray, seed treatment, injections), and high potential toxicity towards insects, neonics appeared as promising substitutes of conventional insecticides, such as organophosphates, carbamates, and synthetic pyrethroid pesticides [1,2], is widely used and available in more than 120 countries [3]. However, neonics can persist in soil, water, and biota [2], being already detected in surface water [5], seed treatments [6], drinking water [7], wastewater treatment plants [8], wetland [8], house dust [9], air [10], and food [11]. Such widespread distribution is worrisome due to their potentially detrimental effects, which have been reported by the scientific community and by regulatory agencies [2,12,13,14]. Within these concerns, the harmful effects of neonics on pollinators is a matter of particular alarm [2,15], as recognized by the European Food Safety Authority (EFSA). Besides the toxicity to honeybees and other non-target organisms, recent evidence demonstrated that neonics are toxic towards mammals [16]. There are a few epidemiological studies confirming the association of human exposure to neonics with neurodevelopmental and neurological detrimental effects [17,18]. Moreover, in 2013, the EFSA Panel on Plant Protection Products and their Residues affirmed that acetamiprid and imidacloprid could damage the human nervous system, which may alter the development of brain and neurons structures affecting memory and learning [2]. Due to all the described concerns, it is of high priority to developing strategies to remove neonics from contaminated matrices, avoiding further environmental contamination and detrimental health impacts.

There are several methods to remove insecticides from aqueous solutions, including oxidation, photocatalysis, electrochemical, and adsorption processes [19]. Among these, adsorption is one of the most promising methods since it is a physicochemical process with several advantages, such as low cost, simplicity, and environment-friendly character if proper materials are applied [19,20]. Due to a panoply of advantages, such as a large specific surface area and high removal efficiency, activated carbon-based materials are the most studied materials for the removal of neonics [19]. For instance, micro and mesoporous activated carbon (TPAC) has been applied as adsorbents to remove acetamiprid [19], whereas granular activated carbon (AC) has been used to remove imidacloprid, thiacloprid, thiamethoxam, and clothianidin [21]. Moreover, a material resulting from the combination of AC with florisil was employed as an adsorbent to remove acetamiprid from several fruits and vegetables [22]. In addition to activated carbon, Dummy Template Surface Molecularly Imprinted Polymers (DMIPs) have been used to adsorb imidacloprid and acetamiprid [23], whereas chitin-derived three-dimensional nanomaterials have been used as adsorbents to remove acetamiprid [24]. Other types of materials include Polar Organic Chemical Integrative Sampler (POCIS), applied to adsorb imidacloprid, acetamiprid, thiacloprid, thiamethoxam, clothianidin, dinotefuran, and nitenpyram [25], and Metal-Organic Frameworks (MOFs) containing thioether-based residues to remove acetamiprid and thiacloprid from aqueous solutions [26]. Potassium hydroxide activated magnetic microporous loofah sponge biochar [27] and potassium hydroxide activated magnetic microporous sugarcane bagasse biochar [28] have also been applied as adsorbents to remove imidacloprid.

Although not investigated up to date, Supported Ionic Liquids (SILs) are materials with interesting properties that could be used to remove insecticides, including neonicotinoids. In SILs, ionic liquids (ILs) are immobilized onto materials, such as silica and polymeric supports [29,30,31,32]. In this last scenario, a diversity of polymers can be applied, giving rise to a class of materials known as poly(ionic liquids) [33]. These materials exhibit both the properties of ionic liquids, such as the designer characteristic and high CO_2_ solubility and polymerization, allowing the increase in their overall performance [34]. In fact, many promising studies have been performed regarding the applicability of poly(ionic liquids) in CO_2_ capture, in which it was shown it is possible to take advantage of the designer nature of the material to improve its performance, i.e., by modifying its structure according to the purpose [35]. SILs exhibit part of the advantages of neat ILs, such as non-volatility, non-flammability, and high tailoring ability through the modification of the materials surface and chemical composition by introducing a diversity of IL chemical structures [31,36]. In SILs, the cation is usually covalently attached to the support, and thus the range of interactions observed in pure ILs is expected to occur in SILs as well [32,37]. This allows the tuning of these materials’ separation or removal performance. IL-modified materials have been applied, for instance, in the extraction and separation of natural compounds from biomass extracts, deoxyribonucleic acid (DNA), and in the removal of active pharmaceutical compounds, as reviewed by Ventura et al. [38], and in the extraction of aromatic compounds from paints (e.g., benzene, toluene, ethylbenzene, and xylenes) [39]. Generally, silica is the most used material due to its low-cost and biocompatibility [40]; however, other materials could also be used, such as polymers and monoliths [41].

Given the proven usefulness of SILs to remove a myriad of compounds, in this work, we investigated, for the first time, their suitability to remove neonics, including imidacloprid, acetamiprid, thiacloprid, and thiamethoxam, from aqueous solutions. SILs were first synthesized and characterized, followed by adsorption studies by accomplishing several kinetic, isotherm, and continuous saturation assays resorting to solid phase extraction (SPE) columns.

## 2. Results and Discussion

### 2.1. Characterization of SILs

The chemical characterization of the synthesized SILs ([Si][N_3222_]Cl, [Si][N_3444_]Cl and [Si][N_3888_]Cl), as well as of silica and of the precursor material ([Si][C_3_]Cl), was initially performed, being also helpful to unveil the adsorption mechanisms involved and to select and design the best SIL. Elemental Analysis was performed for all the synthesized materials to evaluate the success and efficiency of the silica functionalization. Zeta Potential measurements were carried out to determine the point of Zero Charge (PZC) of each SIL, i.e., the pH value at which a solid particle in suspension presents zero net electrical charges on its surface. The percentage weight fraction of carbon, hydrogen, and nitrogen contents found on silica, on the precursor [Si][C_3_]Cl, and on the synthetized SILs [Si][N_3222_]Cl, [Si][N_3444_]Cl, and [Si][N_3888_]Cl, as well as their PZC and bonding amount, are given in Table 1.

The carbon, hydrogen, and nitrogen fraction percentage for the three SILs ranges from 6.4% to 6.7%, from 1.43% to 1.56%, and from 0.06% to 0.24%, respectively. The major element found in all SILs is carbon, followed by hydrogen and nitrogen. These results confirm the presence of organic moieties corresponding to the IL chemical structure in the silica surface. Furthermore, no nitrogen was detected in [Si][C_3_]Cl, the precursor used in the two-step reaction, pointing out the absence of IL organic moieties. The introduction of the cation source onto the SILs structure corresponds to the only source of nitrogen in the synthetic reaction, thus showing the successful functionalization of silica with IL moieties. Compared to silica and to the precursor material, the nitrogen contents of the three SILs are higher, being this increase inversely proportional to the size of the cation alkyl chains. As for the synthesis of the different SILs, a constant volume of the cation source was used; the amount of nitrogen decreases with the increase in the alkyl chain of the cation source. Therefore, the longer the cation alkyl chain length, the lower is the nitrogen percentage in the material, being [Si][N_3222_]Cl the SIL with higher nitrogen content. Overall, the presence of nitrogen, carbon, and hydrogen confirms the success of the covalent attachment of the studied ILs to the silica surface. Our data are in agreement with the literature on the characterization of SILs through elemental analysis [36].

All synthesized SILs exhibit higher PZC values than silica and the precursor, indicating that the surface of SILs is more positively charged. This evidence confirms the presence of the IL cation on the SIL structure and the success of the silica functionalization, being in accordance with the literature [36,42]. Moreover, silica exhibits a PZC of 3.4, which is in agreement with the range of values reported in the literature [43]. The differences in PZC between the three SILs are assumed to be due to their degree of functionalization: if fewer cations are bound to the silica structure, i.e., in those with lower bonding amounts, the PZC value is consequently lower.

Considering the specific surface area of silica gel 60 (S_BET_) of 434.6 m^2^ g^−1^ [36], it is possible to determine the IL bonding amount (BA) to the silica for the precursor material [Si][C_3_]Cl and for each of the SILs under study by applying Equations (1) and (2). According to results given in Table 1, there is a decrease in the bonding amount as follows: [Si][N_3222_]Cl > [Si][N_3444_]Cl > [Si][N_3888_]Cl. This phenomenon could be attributed to the number of moles of each ammine used in the reaction, and as discussed before.

Thermogravimetric analysis (TGA) assays were also performed for the three SILs used in this work ([Si][N_3222_]Cl, [Si][N_3444_]Cl, and [Si][N_3888_]Cl), and the respective degradation temperature profiles are represented in Figures S1–S3 given in Supplementary Materials. According to the results, [Si][N_3222_]Cl and [Si][N_3444_]Cl can be considered thermally more stable since a decay level in their thermogravimetric curve was not verified; however, [Si][N_3888_]Cl exhibits this decay at temperatures of around 250 °C proving its degradation, i.e., mass losses under these conditions. Moreover, since each SIL exhibits in its structure a combination of functionalized material and the precursor material, it is possible to affirm that the higher the functionalization degree, the higher is the thermostability. Regarding the final SIL materials, it should also be noticed that there are still propyl chloride moieties in their structure that did not react with the cationic source. Therefore, it is not possible to apply TGA techniques to determine the functional degree of the materials under study.

Scanning Electron Microscopy (SEM) images representing the morphological characteristics of silica and the synthesized SILs [Si][N_3222_]Cl, [Si][N_3444_]Cl, and [Si][N_3888_]Cl are depicted in Figure S4 in the Supplementary Materials section. The results confirm that all the materials exhibit a rough layer morphology, and no significant differences were found between the synthesized SILs and silica. This could be explained since only a few molecules are bound to the silica in the functionalization processes. This evidence suggests that any difference that could possibly exist in the adsorption efficiencies of the materials in the study must not be related to their morphology.

### 2.2. Adsorption Kinetics

**Figure 1:**
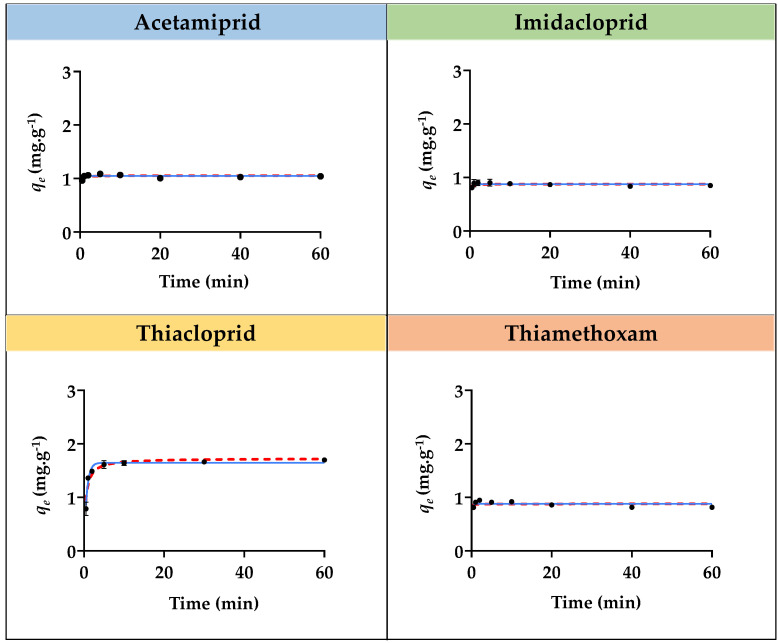
Adsorption kinetic curves for acetamiprid, imidacloprid, thiacloprid, and thiamethoxam with [Si][N_3222_]Cl. The black dots correspond to the experimental data, the continuous blue line corresponds to the fitting of the experimental data with the Pseudo first-order model, and the red dashed line corresponds to the fitting with the Pseudo second-order model.

**Figure 2:**
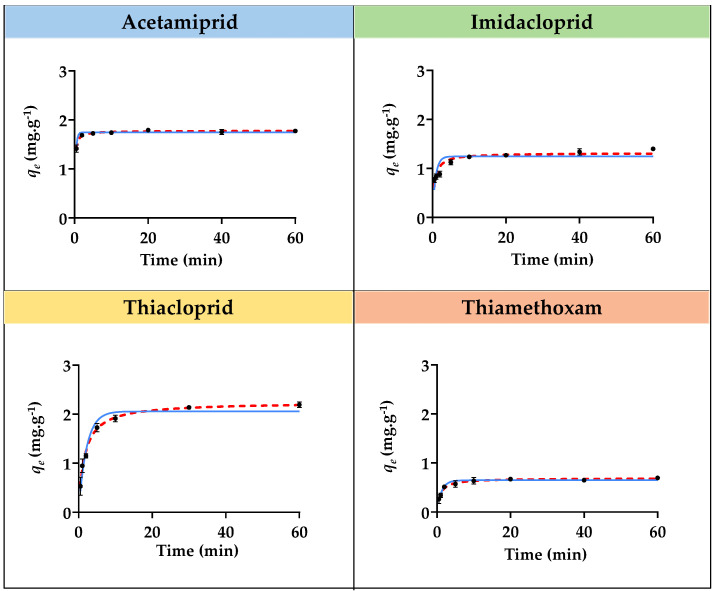
Adsorption kinetics curves for acetamiprid, imidacloprid, thiacloprid, and thiamethoxam insecticides with [Si][N_3444_]Cl. The black dots correspond to the experimental data, the continuous blue line corresponds to the fitting of the experimental data with the Pseudo first-order model, and the red dashed line corresponds to the fitting with the Pseudo second-order model.

**Figure 3:**
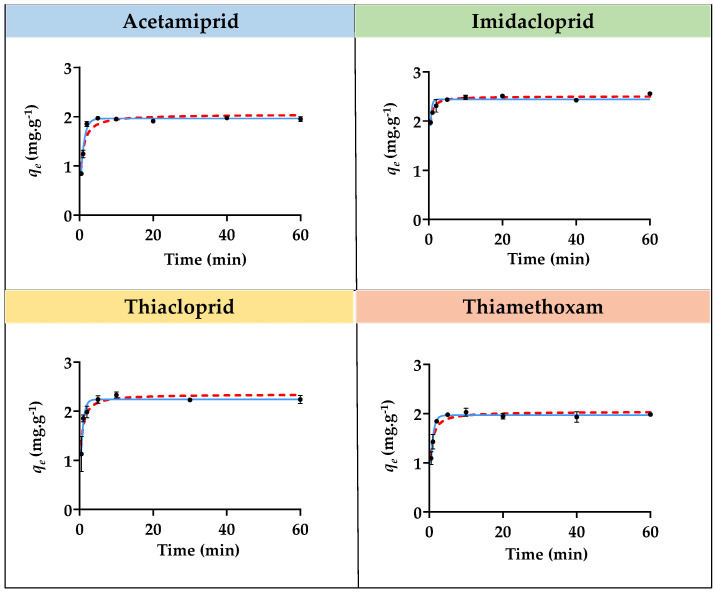
Adsorption kinetics curves for acetamiprid, imidacloprid, thiacloprid, and thiamethoxam insecticides with [Si][N_3888_]Cl. The black dots correspond to the experimental data, the continuous blue line corresponds to the fitting of the experimental data with the Pseudo first-order model, and the red dashed line corresponds to the fitting with the Pseudo second-order model.

The adsorption kinetic studies for the four insecticides with each SIL ([Si][N_3222_]Cl, [Si][N_3444_]Cl, and [Si][N_3888_]Cl) were carried out to evaluate the contact time required to reach equilibrium. The same adsorption studies were carried out with AC, which was used as a benchmark. The adsorption kinetic curves, i.e., the representation of the experimental equilibrium concentration of the sorbate in the solid phase (*q_e_*) values as a function of time for imidacloprid, acetamiprid, thiacloprid, and thiamethoxam with [Si][N_3222_]Cl, [Si][N_3444_]Cl, and [Si][N_3888_]Cl, are depicted in Figure 1, Figure 2 and Figure 3. The graphical representation of the adsorption kinetic curves for AC is given in Figure S5 provided in the Supplementary Materials. The detailed experimental data for all materials and insecticides are compilated in Tables S1–S5, also given in the Supplementary Materials.

The experimental equilibrium concentration of the adsorbate in the solid phase of each insecticide corresponding to each SIL is given in Table 2, decreasing in the following sequence: [Si][N_3888_]Cl > [Si][N_3444_]Cl > [Si][N_3222_]Cl for all the insecticides. The best-identified SIL for the adsorption of these compounds is [Si][N_3888_]Cl, exhibiting higher experimental adsorption capacities (*q_e,exp_*), namely 1.95, 2.48, 2.33, and 2.03 mg g^−1^ for imidacloprid, acetamiprid, thiacloprid, and thiamethoxam, respectively. On the other hand, [Si][N_3222_]Cl and [Si][N_3444_]Cl displayed a better performance to remove thiacloprid, whereas [Si][N_3888_]Cl had a higher performance to remove imidacloprid. The same materials had a lower performance to remove imidacloprid, thiamethoxam, and acetamiprid, respectively. These results show that each SIL is more appropriate to a given insecticide, further indicating that specific interactions between each IL chemical structure and each SIL occur and may rule the adsorption performance of SILs.

As demonstrated by the adsorption kinetic curves (Figure 1, Figure 2 and Figure 3), the equilibrium in all SILs was reached in 5 min or less, being kept at least up to 60 min. This evidence confirms the fast adsorption rate of the three SILs for all insecticides, in contrast with the benchmark AC that requires a period of 40 to 60 min to achieve this stage, thus confirming its slow adsorption nature and in agreement with the literature [19,21]. The graphical representation of the kinetic curves and fitting of the experimental data by AC is provided in Figure S5 in the Supplementary Materials. This fast absorption rate by SILs can be seen as a major advantage when considering their application under the non-batch but yet continuous mode, as will be shown below.

The adsorption efficiencies of [Si][N_3222_]Cl, [Si][N_3444_]Cl, [Si][N_3888_]Cl, and the benchmark AC, at equilibrium and after 10 min of contact with each insecticide were also determined, being given in Table S6 in the Supplementary Materials. The maximum adsorption efficiencies of two of the previously referred SILs, namely [Si][N_3444_]Cl and [Si][N_3888_]Cl, along with silica modified with propyldimethylbutylammonium chloride ([Si][N_3114_]Cl) and silica modified with propylmethyl imidazolium ([Si][C_3_C_1_im]Cl), used in a previous work [36] as adsorbent materials to remove acetylsalicylic acid from aqueous matrices, are provided in Table S7 in the Supplementary Materials. Almeida et al. [42] also used [Si][C_3_C_1_im]Cl as an adsorbent to remove sodium diclofenac from aqueous matrices. Although this imidazolium-based SIL exhibited a remarkable potential for the adsorption of Non-Steroidal Anti-Inflammatory Drugs (NSAIDs) with a maximum equilibrium adsorption capacity of 0.74 mmol of diclofenac *per* gram of adsorbent, it is unable to remove any of the insecticides investigated. Our results are in agreement with those shown by Bernardo et al. [36], who proved that the IL chemical structure influences the final adsorption performance and that the SIL with an aromatic ring, i.e., [Si][C_3_C_1_im]Cl, is not promising to remove acetylsalicylic acid as well. Within all quaternary ammonium-based SILs, [Si][N_3114_]Cl presented the lowest adsorption efficiency to remove the studied insecticides, in contrast to what was verified by Bernardo et al. [36] for acetylsalicylic acid. Based on these results, it is clear that the chemical structure of the IL plays a major role in defining the SIL adsorption performance for each target, reinforcing these materials designer solvents nature.

The adsorption kinetic experimental data were fitted by two kinetic models, namely the Pseudo First-Order and Pseudo Second-Order models, according to Equations (4) and (5). The fitting of the adsorption kinetic experimental data for all insecticides with all SILs with these models is given in Figure 1, Figure 2 and Figure 3, with the obtained parameters being compiled in Table 3. Moreover, the same fitting was applied to the experimental adsorption kinetic data regarding the benchmark AC, which the respective graphical representation is depicted in Figure S5 in the Supplementary Materials. The parameters obtained by fitting the experimental adsorption kinetics data of AC are compilated in Table S8 in the Supplementary Materials.

According to the obtained correlation coefficients (R^2^), the best fitting of the experimental data for all SILs was achieved with the Pseudo Second-Order model, suggesting that the adsorption process is controlled on the solid-liquid interface of the adsorbent. In the case of the benchmark AC, the best fitting for the adsorption kinetic experimental data was achieved with the Pseudo First-Order model, meaning that the adsorption process takes place only on localized sites and no interactions are established with the adsorbed molecules [44]. Overall, the fast adsorption process along with good adsorption efficiencies of SILs could be considered a remarkable advantage for the removal of harmful insecticides, being relevant when foreseeing their application under continuous mode.

### 2.3. Adsorption Isotherms

**Figure 4:**
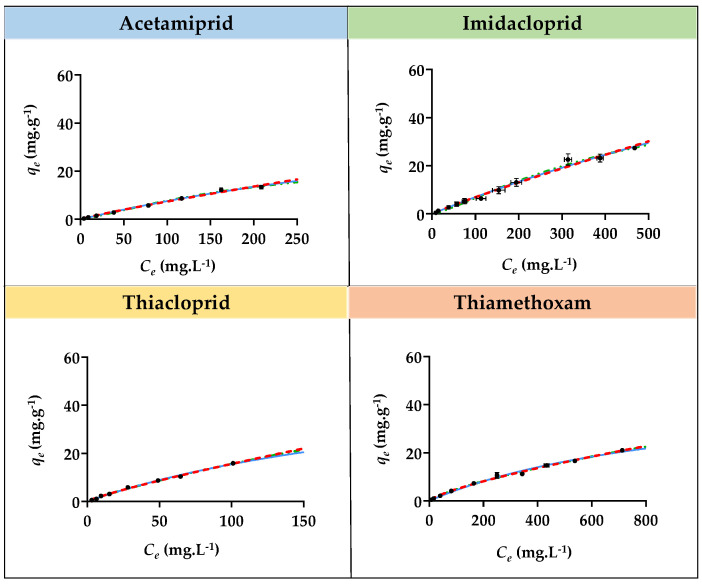
Adsorption isotherms curves for imidacloprid, acetamiprid, thiacloprid, and thiamethoxam insecticides with [Si][N_3222_]Cl. The black dots correspond to experimental data, the continuous blue line corresponds to the fitting of the experimental data with the Langmuir model, the red dashed line corresponds to the fitting with the Freundlich model, and the green dashed and pointed line corresponds to the fitting with the SIPS model.

**Figure 5:**
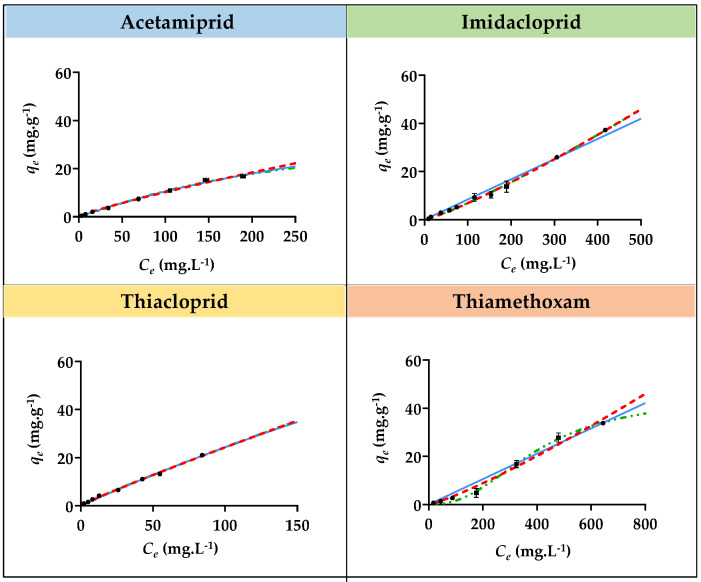
Adsorption isotherms curves for imidacloprid, acetamiprid, thiacloprid, and thiamethoxam insecticides with [Si][N_3444_]Cl. The black dots correspond to experimental data, the continuous blue line corresponds to the fitting of the experimental data with the Langmuir model, the red dashed line corresponds to the fitting with the Freundlich model, and the green dashed and pointed line corresponds to the fitting with the SIPS model.

**Figure 6:**
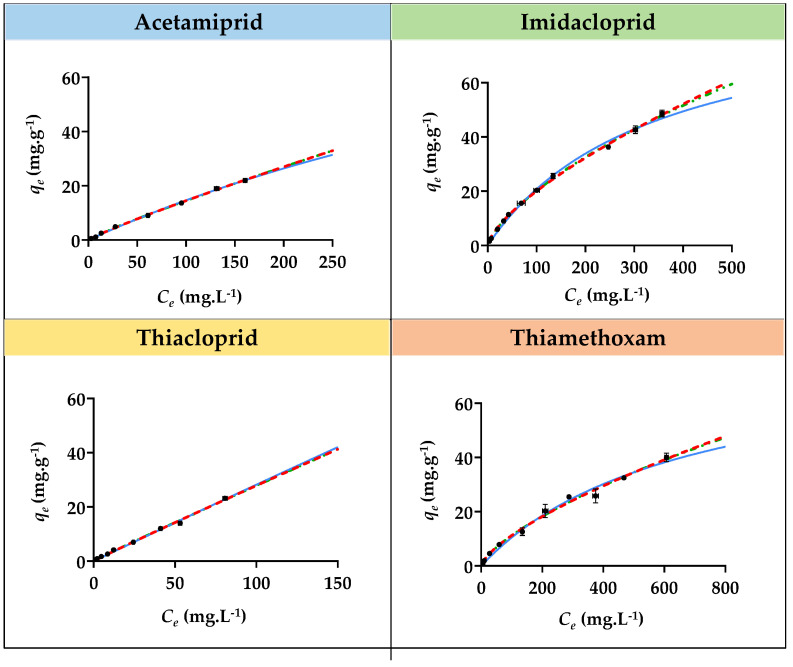
Adsorption isotherms curves for imidacloprid, acetamiprid, thiacloprid, and thiamethoxam insecticides with [Si][N_3888_]Cl. The black dots correspond to experimental data, the continuous blue line corresponds to the fitting of the experimental data with the Langmuir model, the red dashed line corresponds to the fitting with the Freundlich model, and the green dashed and pointed line corresponds to the fitting with the SIPS model.

Adsorption isotherm studies for imidacloprid, acetamiprid, thiacloprid, and thiamethoxam with the three SILs ([Si][N_3222_]Cl, [Si][N_3444_]Cl, and [Si][N_3888_]Cl) and with the benchmark AC were performed, allowing to appraise the relationship between the equilibrium distribution of each insecticide between the liquid and solid phases. The contact time between each SIL and AC with all insecticides was defined as 20 and 120 min, respectively, to ensure that equilibrium was achieved. Additionally, concentrations of each insecticide ranging from 10 to 500 mg L^−1^ were used, except for thiacloprid, where a maximum concentration of 150 mg L^−1^ was used due to its lower water solubility. The graphical representation of the experimental *q_e_* values as a function of the concentration in the equilibrium of sorbent (*C_e_*) for all insecticides with each SIL is depicted in Figure 4, Figure 5 and Figure 6. The graphical representation of the experimental *q_e_* values as a function of *C_e_* for all insecticides with AC is depicted in Figure S6 provided in the Supplementary Materials. The detailed data regarding the equilibrium concentration of all insecticides after adsorption (*C_e_*), the concentration of adsorbate in the solid phase (*q_e_*), and the respective standard deviations (σ) are provided in Tables S9–S16 given in the Supplementary Materials.

The equilibrium adsorption of each insecticide onto all SILs increases with the increase in its initial concentration. However, only a few materials were able to reach saturation or plateau at around the *q_e_* value, corresponding to the equilibrium concentration and saturation of the material. For instance, [Si][N_3222_]Cl seems to be almost achieving a plateau for acetamiprid adsorption. A plateau also seemed to be almost achieved for the adsorption of the same insecticide and for thiamethoxam onto [Si][N_3444_]Cl. Notwithstanding, it was not possible to achieve a saturation or plateau phase for the majority of SILs and insecticides since the *q_e_* values are constantly increasing with the increment of *C_e_*. This impossibility is due to experimental constraints, in which it is not possible to increase even more the insecticide concentration in solution due to its limited water solubility or due to the maximum adsorption capacity of each SIL that does not enable a saturation or plateau to be reached. Even though, based on the gathered results, the relationship between the equilibrium distribution of all insecticides in the study between the liquid and solid phases decreases in the following sequence of SILs: [Si][N_3888_]Cl > [Si][N_3444_]Cl > [Si][N_3222_]Cl, which is in agreement with the adsorption kinetic results. Although a plateau at around the *q_e_* value was not reached, corresponding to the equilibrium concentration and saturation of the material, [Si][N_3888_]Cl allowed the highest maximum adsorption capacity amongst the studied SILs. In contrast to what was verified for the majority of SILs, in the case of benchmark AC (Figure S6 provided in the Supplementary Materials), a saturation or plateau was reached.

The experimental data regarding the adsorption isotherms for all insecticides onto SILs were fitted with the Langmuir, Freundlich, and SIPS isotherm models, given by the Equations (6)–(8). The graphical representation of the experimental data fitting is depicted in Figure 4, Figure 5 and Figure 6, and the parameters obtained from these adjustments are provided in Table 4. The parameters obtained by fitting the experimental adsorption isotherms data of AC are compilated in Table S17 in the Supplementary Materials.

According to the obtained correlation coefficients, it is shown that the best fitting of the experimental data regarding the adsorption isotherms of all SILs for all insecticides is given by the Freundlich isotherm model, meaning that the concentration of adsorbate on the surface increases as the adsorbate concentration increases and, therefore, multiple layers occur instead of a single layer and that the adsorption process is heterogeneous [45]. These results show that interactions between the insecticides occur in SILs. The *n* constant gives information regarding the nature and propensity of the adsorption process. A *n* value ranging from 1 to 10 indicates that the adsorbate is favorably adsorbed on the surface of the adsorbent, and this process has a physical nature, whereas an *n* value lower than 1 indicates that the adsorption process is of a chemical nature. According to the results (Table 4) for all insecticides and SILs, except for the cases of imidacloprid and thiamethoxam adsorption onto [Si][N_3444_]Cl, all *n* values are between 1 to 10, meaning that the adsorbate is favorably adsorbed on the surface of the adsorbent and that the adsorption process has a physical nature. In contrast, for the adsorption isotherms of AC, the best fitting of the experimental data was achieved by the Langmuir model, which curves are represented in Figure S6 in the Supplementary Materials. This evidence indicates that the adsorption of the studied insecticides onto AC takes place by the formation of a monolayer on the outer surface of the adsorbent, where no further adsorption occurs.

Despite not being the best model for the fitting of the experimental data regarding the adsorption isotherms of the insecticides onto SILs, the Langmuir isotherm model can be used to predict the maximum equilibrium concentration (*q_max_*) values. According to the results, the *q_max_* obtained for SILs were significantly higher than the one obtained for AC. For instance, comparing the best identified SIL ([Si][N_3888_]Cl) with AC, *q_max_* of approximately 91.6, 132.7, and 80.93 mg g^−1^ was obtained for imidacloprid, acetamiprid, and thiamethoxam, respectively, whereas lower *q_max_* values for AC of approximately 66, 56, and 46 mg g^−1^ were achieved for the same insecticides. Moreover, assuming the same model for the fitting of the experimental data of the adsorption isotherm of AC for thiacloprid, a lower *q_max_* of approximately 20 mg g^−1^ was predicted; however, for this case, a comparison with [Si][N_3888_]Cl could not be established since a non-proper fitting of the experimental data was achieved with the Langmuir model. Notwithstanding, the latter *q_max_* obtained for AC was significantly lower than the one achieved for [Si][N_3222_]Cl of 58.5 mg g^−1^. These results confirm the high adsorption capacity of SILs, reinforcing their potential as adsorbent materials to remove harmful neonics from aqueous matrices.

Although the best fitting of the experimental data regarding the adsorption isotherms was not achieved with the SIPS model, it is possible to use the gathered data to address the heterogeneity character of the adsorption process by analyzing the *n_S_* parameter for all SILs. When *n_S_* is equal to 1, the SIPS isotherm experiences an approximation to the Langmuir isotherm, meaning that a homogenous adsorption process could occur, whereas when this constant deviates from the unity, this model tends to experience an approximation to the Freundlich isotherm model predicting the presence of a heterogeneous adsorbent surface. According to the results given in Table 4, the majority of SILs have *n_S_* constant values deviated from 1, confirming the approximation of the experimental data to the Freundlich isotherm model and, therefore, the heterogeneity of the process.

There are some important aspects to be discussed regarding the interactions that may take place between SILs and insecticides to better understand the adsorption mechanism involved. Possible interactions that might occur during the adsorption process are electrostatic interactions; however, all the experiments were performed using insecticides solutions with a pH of ca. 6.0. Under these conditions, and according to the speciation profile of the four insecticides [46], these compounds are neutral, indicating that the adsorption ability is not related to their charge or is governed by electrostatic interactions. For all insecticides, the adsorption capacity of SILs decreases according to the following sequence: [Si][N_3888_]Cl > [Si][N_3444_]Cl > [Si][N_3222_]Cl. In other words, the longer the alkyl chains of the SIL cation, the higher is the SIL adsorption capacity. Therefore, hydrophobic interactions seem to be the main drivers in the favorable adsorption mechanism between SILs and insecticides.

### 2.4. Continuous Removal of Insecticide by SPE

**Figure 7:**
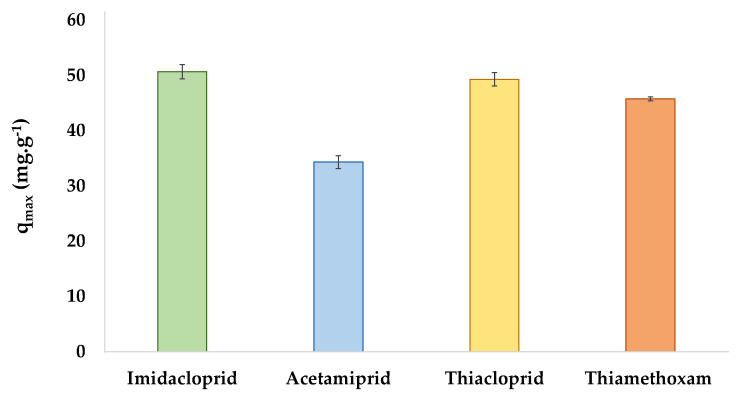
Maximum adsorption capacities were obtained from the continuous experiment in an SPE column using [Si][N_3888_]Cl as adsorption material.

To unveil the maximum adsorption capacity of the best identified SIL, i.e., [Si][N_3888_]Cl, and to prove its use under continuous mode and more close to a real scenario while taking advantage of the high fast adsorption rate, a continuous removal assay using an SPE column packed with SIL was performed. The maximum adsorption capacities obtained from column saturation experiments using [Si][N_3888_]Cl as adsorbent are depicted in Figure 7.

It is noticeable that [Si][N_3888_]Cl exhibits a high maximum adsorption capacity for all insecticides, ranging from approximately 34.28 to 50.65 mg g^−1^, and decreasing in the following sequence: imidacloprid > thiacloprid > thiamethoxam > acetamiprid. The maximum adsorption capacity for all insecticides with [Si][N_3888_]Cl and their respective standard deviation are given in Table S18 in the Supplementary Materials. The experimental maximum adsorption capacities obtained for the best identified SIL are similar to those obtained from the adjustment of the experimental adsorption data of these insecticides onto AC with the Langmuir isotherm model. Still, one of the great advantages of using SILs is related to the relatively short time to reach equilibrium. Moreover, previous works with imidazolium-based SILs already demonstrated the applicability of these materials under continuous conditions [42], which reinforces the potential of these materials to be used to remove pollutants in real scenarios, and therefore, similar results are expected for the SILs studied in this work.

Several materials have been reported in the literature as adsorbents for a variety of insecticides, for instance, porous AC [19], granular AC [21], a combination of AC and florisil [22], DMPIS [23], chitin-derived three-dimensional nanomaterials [24], MOFs [26], POCIS [25], potassium hydroxide activated magnetic microporous loofah sponge biochar [27], and potassium hydroxide activated magnetic sugarcane bagasse biochar [28]. Mohammad et al. [19] studied the application of micro- and mesoporous activated carbon (TPAC) to remove acetamiprid, in which a maximum adsorption capacity of 35.7 mg g^−1^ was achieved at the equilibrium time of 240 min. On the other hand, Webb et al. [21] demonstrated that granular AC has a *q_max_* ranging from 60 to 150 mg g^−1^ for imidacloprid, thiacloprid, thiamethoxam, and clothianidin at a high equilibrium time, namely 16 days, confirming the low adsorption rate of granular AC. Nawaz et al. [22] used AC combined with florisil to remove acetamiprid, in which adsorption efficiencies ranging from 82% to 90% were obtained. Removal efficiencies higher than 90% were achieved by DMIPs in the adsorption of imidacloprid and acetamiprid after 150 min, with a *q_max_* of 42.05 and 22.99 mg g^−1^, respectively [23]. Cheng et al. [24] used chitin-derived three-dimensional nanomaterials to adsorb acetamiprid, achieving a *q_max_* between 79.4 and 84.7 mg g^−1^. POCIS was applied to adsorb solutions with a low concentration (100 µg L^−1^) of various neonics, including imidacloprid, acetamiprid, thiacloprid, thiamethoxam, clothianidin, dinotefuran, and nitenpyram, and adsorption efficiencies ranging from 66 to 95% were obtained after 24 h [25]. In contrast, AC was able to obtain significantly lower adsorption efficiencies ranging from 5 to 48% for the same neonics [25]. Negro et al. [26] showed the ability of MOFs to adsorb acetamiprid and thiacloprid from aqueous solutions of 100 mg L^−1^, in less than 30 s. The achieved removal efficiencies were 60% and 30% for imidacloprid and thiamethoxam, respectively, and the *q_max_* for all the pesticides in the study ranged from 400 and 500 mg g^−1^. Ma et al. [27] used potassium hydroxide activated magnetic microporous loofah sponge biochar to remove imidacloprid, registering a maximum adsorption capacity of 738 mg g^−1^, whereas when a potassium hydroxide activated magnetic microporous sugarcane bagasse biochar was used as adsorbent for the same insecticide, a lower maximum adsorption capacity of 313 mg g^−1^ was achieved [28]. From all the reported materials, SILs are highly competitive in what concerns their adsorption performance for insecticides, with a major advantage of having a significantly high adsorption rate. By comparing SILs with various AC, typically used as a benchmark, it is possible to confirm that SILs present higher maximum adsorption capacities that are achieved in a significantly lower time of exposure to the insecticide. Only MOFs have been reported with a lower adsorption rate. However, SILs exhibit several advantages over MOFs, for instance their lower cost and easy production.

Sadaria et al. [8] determined the occurrence of several insecticides, including acetamiprid, imidacloprid, thiacloprid, and thiamethoxam in contaminated water from wastewater and wetland treatment, with reported concentrations of 2.1, 48.2, <0.9, and <0.3 ng L^−1^, respectively. Under ideal conditions, and based on the *q_max_* values obtained under the continuous mode, it is possible to estimate the volume (m^3^) of contaminated water that could be treated with 1 g of the best identified SIL, i.e., [Si][N_3888_]Cl. Under ideal conditions, 1 g of SIL could treat 1 × 10^6^, 2 × 10^7^, 5 × 10^7^, and 2 × 10^8^ m^3^ of water contaminated with imidacloprid, acetamiprid, thiacloprid, and thiamethoxam, respectively. These extremely high amounts of wastewater and wetland that could be treated reinforce the potential of SILs in the field of remediation of contaminated matrices with insecticides.

## 3. Materials and Methods

To achieve the main purpose of this work, i.e., to appraise and show the applicability of SILs as adsorbent materials to remove neonics from aqueous solutions, two major steps were conducted: (1) synthesis and characterization of SILs; (2) adsorption studies (kinetic, isotherm and under continuous mode) of insecticides onto these materials. The reagents used to perform the synthesis of SILs were (3-chloropropyl)trimethoxysilane (CAS 2530-87-2) and tributylamine (CAS 102-82-9) with 98% and >99% purity, respectively, both acquired from Acros Organics (United Kingdomn and Germany, respectively); trioctylamine (CAS 1116-76-3) with >98% purity and purchased from Fluka (Tokyo, Japan); and triethylamine (CAS 121-44-8) of HPLC grade acquired from Fisher Chemical (Illkirch Cedex, France). Hydrochloric acid (CAS 7647-01-0), classified as ACS reagent with 37% purity with >99.8% purity was acquired from Fluka (Austria), and toluene (CAS 108-88-3) with 99.8% purity, were purchased from Fluka (London, UK). HPLC grade ethanol (CAS 64-17-5) with 99.8% purity and methanol (CAS 67-56-1) with ≥99.9% of puritywere both acquired from Fluka (London, UK). The starting silica gel particles (60Å 0.2–0.5 mm) (CAS 7631-86-9) were acquired from Merck (Darmstadt, Germany). To perform adsorption studies, aqueous solutions were prepared using distilled water and several neonics insecticides, namely imidacloprid (CAS 138261-41-3) with ≥98% purity andthiamethoxam (CAS 153719-23-4) with ≥98% purity, thiacloprid (CAS 111988-49-9) with ≥98% purity both purchased from Sigma Aldrich (Buchs, Switzerland), and acetamiprid (CAS 190604-92-3) with ≥99% purity acquired from Sigma Aldrich (Darmstadt, Germany). 

### 3.1. SILs Preparation

**Figure 8:**
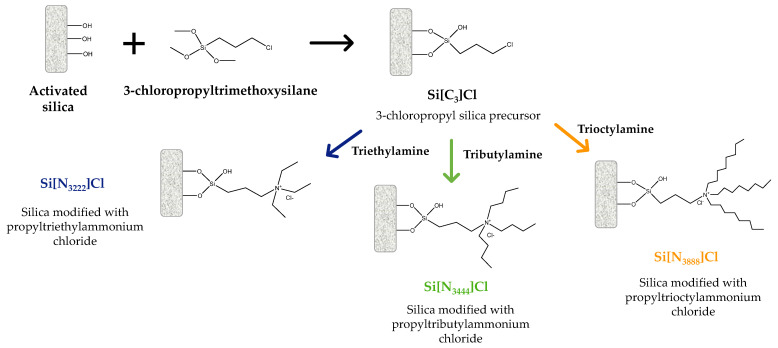
Overview of the synthetic route of SILs, along with the chemical structures of the IL attached to the silica surface and respective names and abbreviations.

The SILs investigated correspond to silica modified with propyltriethylammonium chloride ([Si][N_3222_]Cl), silica modified with propyltributylammonium chloride ([Si][N_3444_]Cl), and silica modified with propyltrioctylammonium chloride ([Si][N_3888_]Cl), which were synthesized following the method described by Bernardo et al. [36]. Figure 8 depicts the synthetic route for SILs preparation, along with the chemical structures of the ILs in the silica surface and respective names and abbreviations.

To prepare SILs, 5 g of silica, previously activated with hydrochloric acid over 24 h, were mixed with 50 mL of toluene and then 5 mL of (3-chloropropyl)triethoxysilane was added. This suspension was left in reflux at 105 °C under magnetic agitation for 24 h. After that, the suspension was filtered using a vacuum glass filter, as soon as the temperature dropped to values close to room temperature, followed by a washing step with 100 mL of toluene, 200 mL of ethanol: water (1:1, *v*/*v*), 500 mL of distilled water and 100 mL of methanol. The resultant materials were dried at 50 °C for at least 24 h. The obtained dried 3-chloropropyl silica precursor ([Si][C_3_]Cl) was added to 5 mL of the compound acting as the cation source (each ammine) in 50 mL of toluene. This suspension was left in reflux at 105 °C under magnetic agitation over 24 h. Afterward, the suspension was filtered and washed with 100 mL of toluene, 350 mL of methanol, 300 mL of distilled water, and 150 mL of methanol followed by a drying step at 50 °C for at least 24 h.

### 3.2. Quantification of Neonic Insecticides

The quantification of thiacloprid, acetamiprid, imidacloprid, and thiamethoxam in the aqueous solutions was performed by Ultraviolet-Visible (UV-Vis) spectroscopy, using a Shimadzu UV-1800, Pharma-Spec UV-Vis equipment (Kyoto, Japan), and quartz cells with 1 × 1 cm dimensions. A calibration curve for each compound was prepared at the respective maximum absorbance wavelength, which was defined as 270, 234, 251, and 242 nm for imidacloprid, acetamiprid, thiamethoxam, and thiacloprid, respectively. For each sample, at least three replicates were read.

### 3.3. Characterization of SILs

#### 3.3.1. Elemental Analysis

The elemental composition of each SIL was determined by elemental analysis using Truspec 630-200-200 equipment (Marietta, GA, USA). To perform this analysis, it was used a sample of approximately 2 mg, a combustion furnace temperature of 1075 °C, and an afterburner temperature of 850 °C. Carbon and hydrogen elements were detected by infrared absorption, whereas nitrogen was determined through thermal conductivity.

#### 3.3.2. Point of Zero Charge (PZC)

The point of zero charge was determined by measuring the zeta potential of aqueous suspensions of the materials in a wide range of pH values. Aqueous solutions of NaOH and HCl 0.01 M were used to adjust the pH. The results were acquired using a Malvern Zetasizer Nano ZS (Malvern Instruments Ltd., Malvern, UK) equipment at room temperature and a capillary zeta cell.

#### 3.3.3. Bonding Amount (BA)

The IL bonding amount to each material was determined by considering the specific surface area of silica gel 60 (S_BET_) of 434.6 m^2^ g^−1^, determined in work by Bernardo et al. [36]. For the precursor material, i.e., Si[C_3_]Cl–cf. Figure 8, this parameter was obtained using the following equation:

(1) $${\mathrm{BA}}_{\mathrm{Si}\left[C3\right]\mathrm{Cl}}=\frac{\frac{\%C}{3\times M\left(C\right)}}{S_{\mathrm{BET}}}$$

where %C corresponds to the percentage weight fraction of carbon present in the material, M(C) is the molar weight of carbon, which is further multiplicated by three to represent the three carbons present in the molecule, and S_BET_ is the specific surface area of silica.

The bonding amount of IL in each SIL was obtained using the following equation:

(2) $${\mathrm{BA}}_{\mathrm{SIL}}=\frac{\frac{\%N}{1\times M\left(N\right)}}{S_{\mathrm{BET}}}$$

where %N corresponds to the percentage weight fraction of nitrogen present in each SIL, M(N) is the molar weight of nitrogen, which is further multiplicated by one to represent the only nitrogen present in the molecule, and S_BET_ is the surface area of silica.

#### 3.3.4. Thermogravimetric Analysis

Degradation temperatures were obtained using a Setsys Evolution 1750 (SETARAM, France) instrument under nitrogen atmosphere at 10 K min^−1^ (precision: temperature ± 0.01 K; mass ± 0.01 mg).

#### 3.3.5. Scanning Electron Microscopy (SEM)

To perform the Scanning Electron Microscope analysis of each of the prepared SILs, it was used a Hitachi SU-70 instrument (Tokyo, Japan). A sample of each SIL was placed in a piece of carbon tape, and, to further increase the conductivity, a thin carbon film was deposited.

### 3.4. Adsorption Studies

To evaluate the efficiency of the adsorptive process with SILs, adsorption kinetic studies were performed using aqueous solutions of each insecticide with a concentration of 20 mg L^−1^, except for thiacloprid in which a concentration of 15 mg L^−1^ was used due to its lower water solubility. 5 mL of each of the prepared insecticide solutions were placed in contact with 25 mg of the synthesized SILs, namely [Si][N_3222_]Cl, [Si][N_3444_]Cl, and [Si][N_3888_]Cl. These suspensions were kept in agitation in an orbital shaker at 25 °C and 250 rpm. Samples from each solution were withdrawn at different times, namely 1, 2, 5, 10, 20, 30, 45, and 60 min, and then after each subsequent hour until a plateau was reached. An aliquot of each suspension was centrifuged for 2 min at 12,000 rpm for the total deposition of the solid. After centrifugation, the amount of insecticide in the aqueous solution was quantified by UV-Vis spectroscopy. For each time point, at least three replicates were performed. For the adsorption isotherm studies, the mass of SILs was maintained constant at 25 mg for all insecticides, except for thiacloprid in which 15 mg was used. The concentration of insecticides varied from 10 mg L^−1^ to 500 mg L^−1^; however, for thiacloprid, due to its lower water solubility, the maximum concentration used was 150 mg L^−1^. The method applied is the same as depicted in the kinetic experiments, using 5 mL of each test solution in separate flasks containing the SILs. After 20 min of contact, samples were centrifuged for 2 min at 12,000 rpm. The supernatant was collected, and the concentration of insecticides was quantified, as previously described. The same protocol in the kinetic and isotherm studies was applied using activated carbon instead of SILs for comparison purposes. However, since the adsorption rate of AC is significantly lower than the one obtained with SILs, 120 min of contact was performed instead of the 20 min employed for SILs.

Adsorption kinetic studies allow the evaluation of the adsorption uptake over time at a constant concentration and temperature. In contrast, adsorption isotherm studies are applied to establish the relationship between the quantity of sorbate adsorbed and the quantity of adsorbate under equilibrium conditions at a constant temperature, giving information on the adsorption mechanism involved. The equilibrium concentration of the sorbate in the solid phase (*q_e_*) was determined by the following equation:

(3) $$q_{e}=\frac{\left(C_{0}\times C_{e}\right)\times V}{w}$$

where *w* corresponds to the mass of SIL used (g), *V* is the volume of the sorbate solution (L), *C*_0_ (mmol L^−1^) is the initial concentration, and *C_e_* (mmol L^−1^) is the equilibrium concentration of the adsorption process.

The adsorption kinetic usually follows one of the two following models, namely the Pseudo First-Order or Pseudo Second-Order models. The first model is described by the following equation [44]:

(4) $$\frac{dq_{t}}{dt}=k_{1}\times \left(q_{e}-q_{t}\right)$$

where *t* is the time (min), *q_e_* is the amount of sorbent bound to the sorbate at equilibrium (mmol g^−1^), *q_t_* is the amount of sorbent bound to the sorbate at a given time (mmol g^−1^), and *k*_1_ (min^−1^) corresponds to the Pseudo First-Order constant [44].

The second model describes the system behavior throughout the sorption range, including the adsorption capacity of the solid, and it can be defined through the following equation:

(5) $$\frac{dq_{t}}{qt}=k_{2}\times {\left(q_{e}-q_{t}\right)}^{2}$$

where *k*_2_ corresponds to the Pseudo Second-Order constant [47,48].

Regarding the adsorption isotherms, they may follow the Langmuir or the Freundlich isotherm models. The first model assumes that the adsorption occurs at a specific point on the material surface and that the sorbate molecules do not interact with each other, resulting in a maximum of adsorption corresponding to the formation of a monolayer. This model is defined through the following equation:

(6) $$q_{e}=\frac{q_{max}\times B\times C_{e}}{1+B\times C_{e}}$$

where *C_e_* is the concentration in the equilibrium of sorbent (mmol L^−1^), *q_e_* is the equilibrium concentration of the sorbent in the solid phase (mmol g^−1^), *B* (L mmol^−1^) is the Langmuir isotherm constant, and *q_max_* is the maximum capacity of the monolayer [47,48].

The Freundlich isotherm model assumes that as the adsorbate concentration increases, the concentration of adsorbate on the adsorbent surface increases as well, being described by the following equation:

(7) qe=Kf×Ce1n

where *K_f_* and *n* are the Freundlich isotherm constants, in which *n* values between 1 and 10 indicate favorable adsorption, being related to the non-linearity of the model [48].

Additionally, the SIPS isotherm model results from a combination of the Langmuir and Freundlich isotherms models, being expressed using the following equation [49]:

(8) $$q_{e}=\frac{q_{m}{\left(K_{s}C_{e}\right)}^{1/n_{S}}}{1+{\left(K_{S}C_{e}\right)}^{1/n_{S}}}\;$$

where *K_S_* (L mg^−1^) corresponds to the equilibrium constant related to the bonding energy adsorption or binding affinity and *n_S_* corresponds to the adsorption intensity constant describing the surface heterogeneity. When *n_S_* is equal to 1, the SIPS isotherm experiences an approximation to the Langmuir isotherm, predicting the occurrence of a homogenous adsorption process. However, when *n_S_* deviates from the unity, this model tends to experience an approximation to the Freundlich isotherm model predicting the presence of a heterogeneous adsorbent surface [49].

The experimental data regarding the adsorption kinetics and isotherms for all insecticides and adsorbent materials were fitted using the GraphpadPrism7 software (San Diego, CA, USA).

### 3.5. Continuous Saturation Resorting to an SPE Column

To study the continuous saturation using a SPE column, an Econo-Pac^®^ Chromatography Column from BioRad (Hercules, CA, USA) was packed with 200 mg of SIL. A concentrated solution of each pesticide, namely 637.4 mg L^−1^ for imidacloprid, 533.2 mg L^−1^ for acetamiprid, 154.1 mg L^−1^ for thiacloprid, and 961.0 mg L^−1^ for thiamethoxam, was then passed under continuous mode to saturate the SIL, assisted with a peristaltic pump. After going through the SIL, aliquots of 5 mL of each solution were collected along time, and for all of them, the amount of pesticide was quantified to determine the amount of pesticides absorbed by the SIL material under continuous mode. When the concentration of the solution that went through the SIL was similar to the concentration of the solution added to the column, the column saturation was considered to be reached.

## 4. Conclusions

In this work, the removal of four neonicotinoid insecticides, namely imidacloprid, acetamiprid, thiacloprid, and thiamethoxam, using SILs as alternative adsorbent materials was studied. SILs were synthesized and characterized and then applied as adsorbent materials in kinetic and isotherm assays. The results provided by elemental analysis proved the silica functionalization with IL moieties. All SILs presented more positively charged surfaces according to their higher PZC values, confirming the presence of the IL cation on the SIL structure and the success of the silica functionalization.

For all studied insecticides, the equilibrium concentration of the adsorbate in the solid phase decreases in the following sequence: [Si][N_3888_]Cl > [Si][N_3444_]Cl > [Si][N_3222_]Cl. The best-identified SIL for the adsorption of all insecticides was [Si][N_3888_]Cl, exhibiting higher experimental adsorption capacities (*q_e,exp_*). The saturation of SILs was reached in 5 min or less, showing their fast adsorption rates towards all insecticides, in contrast with the benchmark AC that requires a period of 40 to 60 min. The faster adsorption process along with good adsorption efficiencies could be considered a remarkable advantage of SILs as adsorbent materials for the removal of harmful insecticides, being relevant when foreseeing their application under continuous mode in a real scenario. The best fitting of the adsorption kinetic experimental data for all insecticides onto all SILs was achieved by the Pseudo Second-Order model, revealing that the adsorption process is controlled on the liquid-solid interface in the adsorbent. In contrast, the best fitting for the adsorption kinetic experimental data of all insecticides onto AC was achieved by the Pseudo First-Order model, meaning that the adsorption process takes place only on localized sites and that no interactions are established between the adsorbed molecules.

From the adsorption isotherm studies, it was shown that the equilibrium adsorption of each insecticide onto all SILs increases with the increase in its initial concentration, in which only a few materials were able to reach saturation. Even so, it was possible to conclude that the relationship between the equilibrium distribution of all insecticides in the study between the liquid and solid phases decreases in the following sequence of SILs: [Si][N_3888_]Cl > [Si][N_3444_]Cl > [Si][N_3222_]Cl. The best fitting of the adsorption isotherm experimental data of all insecticides onto all SILs is given by the Freundlich isotherm model, meaning that the concentration of adsorbate on the surface increases as the adsorbate concentration increases and, therefore, multiple layers could occur instead of a single layer of insecticide. In contrast, the best fitting of the adsorption isotherm experimental data of AC was achieved by the Langmuir model, indicating that the adsorption of the insecticides takes place by the formation of a monolayer on the outer surface of the adsorbent.

Among the studied SILs, [Si][N_3888_]Cl allowed the highest maximum adsorption capacity, being finally used in column saturation assays or SPE under continuous mode. The material maximum adsorption capacity decreases in the following sequence of insecticides: imidacloprid > thiacloprid > thiamethoxam > acetamiprid. These values range from approximately 34.28 to 50.65 mg g^−1^, being competitive with other materials reported in the literature. Overall, the enhanced adsorption capacity of SILs for a broad diversity of neonics coupled to their high adsorption rate here demonstrated reinforces the usefulness of these materials to remove neonics from aqueous matrices. Based on real reported values of the occurrence of imidacloprid, acetamiprid, thiacloprid, and thiamethoxam on contaminated wastewater plant treatment and water from wetland and under ideal conditions, 1 g of SIL could treat 1 × 10^6^, 2 × 10^7^, 5 × 10^7^, and 2 × 10^8^ L of water contaminated with imidacloprid, acetamiprid, thiacloprid, and thiamethoxam, respectively. SILs are thus promising candidates to be further investigated at a scaled-up level to prevent the introduction of neonics into the ecosystems and reduce their detrimental effects on the environment and human health.

## Figures and Tables

**Table 1:** Percentage weight fraction of carbon, hydrogen, and nitrogen obtained by Elemental analysis, along with the PZC and bonding amount for silica, [Si][C_3_]Cl, and the three SILs [Si][N_3222_]Cl, [Si][N_3444_]Cl, and [Si][N_3888_]Cl.

SIL	Elemental Analysis			PZC	Bonding Amount (µmol m^2^)
	% Carbon	% Hydrogen	% Nitrogen		
Silica	-	-	-	3.4 ± 0.2	-
[Si][C_3_]Cl	4.637 ± 0.486	1.394 ± 0.047	-	4.2 ± 0.1	2.964
[Si][N_3222_]Cl	6.724 ± 0.565	1.563 ± 0.054	0.276 ± 0.013	9.0 ± 0.2	0.454
[Si][N_3444_]Cl	6.379 ± 0.638	1.427 ± 0.091	0.139 ± 0.009	6.0 ± 0.8	0.228
[Si][N_3888_]Cl	6.426 ± 0.473	1.432 ± 0.097	0.060 ± 0.011	5.5 ± 0.3	0.099

**Table 2:** Experimental equilibrium concentration of the adsorbate in the solid phase (*q_e_*, experimental) of acetamiprid, imidacloprid, thiacloprid, and thiamethoxam for [Si][N_3222_]Cl, [Si][N_3444_]Cl, and [Si][N_3888_]Cl.

Insecticide	*q_e_*, Experimental (mg g^−1^)		
	[Si][N_3222_]Cl	[Si][N_3444_]Cl	[Si][N_3888_]Cl
Acetamiprid	1.067	1.739	1.954
Imidacloprid	0.884	1.237	2.479
Thiacloprid	1.640	1.911	2.332
Thiamethoxam	0.918	0.636	2.027

**Table 3:** Adsorption parameters obtained from the fitting of adsorption kinetic experimental data with the Pseudo First-Order and Pseudo Second-Order models, and the respective correlation coefficients for imidacloprid, acetamiprid, thiacloprid, and thiamethoxam with [Si][N_3222_]Cl, [Si][N_3444_]Cl, and [Si][N_3888_]Cl.

Pesticide	Material	Pseudo First-Order			Pseudo Second-Order			Equilibrium Time (min)
		q_t_ (mg g^−1^)	k_1_ (min^−1^)	R^2^	q_t_ (mg g^−1^)	k_2_ (min^−1^)	R^2^	
Imidacloprid	[Si][N_3222_]Cl	0.876	0.521	0.456	0.876	49.770	0.158	2
	[Si][N_3444_]Cl	1.244	1.197	0.643	1.314	1.405	0.676	10
	[Si][N_3888_]Cl	2.439	3.019	0.772	2.503	2.818	0.961	5
Acetamiprid	[Si][N_3222_]Cl	1.048	5.012	0.606	-	27.810	0.384	1
	[Si][N_3444_]Cl	1.745	3.315	0.932	1.776	4.459	0.984	2
	[Si][N_3888_]Cl	1.963	1.113	0.985	2.049	0.884	0.910	5
Thiacloprid	[Si][N_3222_]Cl	1.648	1.454	0.963	1.730	1.378	0.918	5
	[Si][N_3444_]Cl	2.057	0.467	0.955	2.242	0.278	0.993	20
	[Si][N_3888_]Cl	2.244	1.492	0.957	2.349	1.073	0.917	5
Thiamethoxam	[Si][N_3222_]Cl	0.881	5.236	0.186	0.878	91.840	0.020	1
	[Si][N_3444_]Cl	0.648	0.804	0.945	0.690	0.167	0.981	10
	[Si][N_3888_]Cl	1.967	1.444	0.977	2.039	1.273	0.939	5

**Table 4:** Adsorption parameters obtained from the fit of adsorption isotherms experimental data isotherms with the Freundlich, Langmuir, and SIPs models, and the respective correlation coefficients for imidacloprid, acetamiprid, thiacloprid, and thiamethoxam with [Si][N_3222_]Cl, [Si][N_3444_]Cl, and [Si][N_3888_]Cl.

Pesticide	Material	Freundlich			Langmuir			SIPS				
		*k_f_* (mg g^−1^)	*n*	R^2^	*q_max_* (mg g^−1^)	B (L mg^−1^)	R^2^	*q_max_* (mg g^−1^)	*K_S_* (L mg^−1^)	*n*	R^2^	*q_emax_* (mg g^−1^)
Imidacloprid	[Si][N_3222_]Cl	0.103	1.094	0.987	-	4.749 × 10^−4^	0.989	57.640	0.002	1.266	0.991	27
	[Si][N_3444_]Cl	0.030	0.848	0.997	No Fit			No Fit				37
	[Si][N_3888_]Cl	0.829	1.447	0.999	91.570	0.003	0.995	-	1.737 × 10^−4^	0.737	0.999	48
Acetamiprid	[Si][N_3222_]Cl	0.125	1.131	0.991	61.710	0.001	0.993	-	0.004	1.217	0.995	13
	[Si][N_3444_]Cl	0.211	1.185	0.993	61.180	0.002	0.995	-	0.004	1.124	0.996	17
	[Si][N_3888_]Cl	0.234	1.116	0.999	132.700	0.001	0.998	No Fit				22
Thiacloprid	[Si][N_3222_]Cl	0.322	1.186	0.996	58.500	0.004	-	No Fit				16
	[Si][N_3444_]Cl	0.342	1.080	0.997	No Fit			No Fit				21
	[Si][N_3888_]Cl	0.323	1.033	0.995	No Fit			No Fit				23
Thiamethoxam	[Si][N_3222_]Cl	0.161	1.350	0.995	48.860	0.001	0.992	-	9.842 × 10^−5^	0.789	0.995	21
	[Si][N_3444_]Cl	0.0162	0.841	0.982		No Fit		45.110	0.002	2.383	0.996	34
	[Si][N_3888_]Cl	0.453	1.434	0.992	80.930	0.001	0.988	No Fit				40

## Data Availability

Not applicable.

## Supplementary Materials

The following are available online at https://www.mdpi.com/article/10.3390/ijms23062989/s1.
