# Supplementary material for: High Performance of Ionic-Liquid-Based Materials to Remove Insecticides
Source: Int J Mol Sci. 2022 Mar 10;23(6):2989. doi: 10.3390/ijms23062989 (PMC8950405; doi:10.3390/ijms23062989)
Supplement: Supplementary file 1 [file ijms-23-02989-s001.zip › ijms-1559789-supplementary.pdf]

Supplementary Material

High Performance of Ionic-Liquid-Based Materials in Removing Neonicotinoid Insecticides

Rafael Francisco [1], Catarina Almeida[1], Ana C. A. Sousa[2], Márcia C. Neves[1] and Mara G. Freire[1*]

[1] CICECO-Aveiro Institute of Materials, Department of Chemistry, University of Aveiro, 3810-193 Aveiro, Portugal; rafaelfrancisco@ua.pt; ac.almeida@ua.pt; mcneves@ua.pt; maragfreire@ua.pt
[2] Department of Biology and Comprehensive Health Research Centre (CHRC), University of Évora, 7004-554 Évora, Portugal; acsousa@uevora.pt
* Correspondence: maragfreire@ua.pt

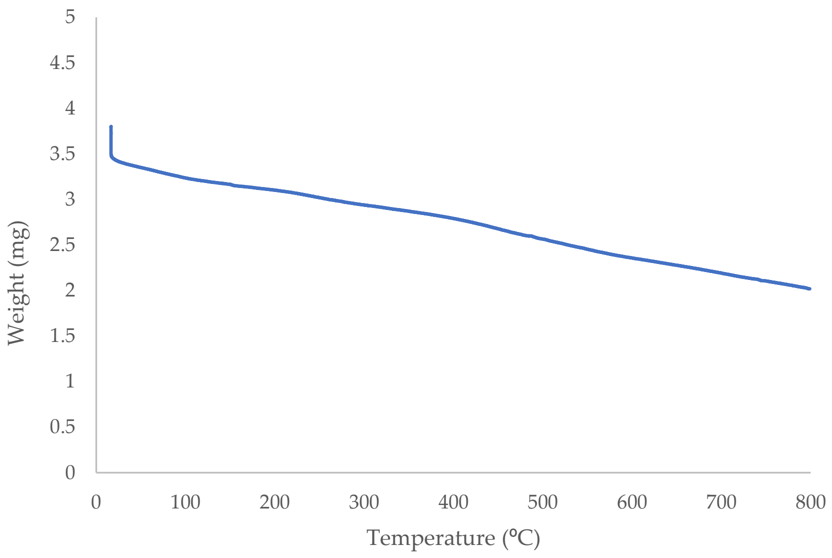

Figure S1. Degradation temperatures of a [Si][N_{3222}]Cl sample by Thermogravimetric analysis (TGA).

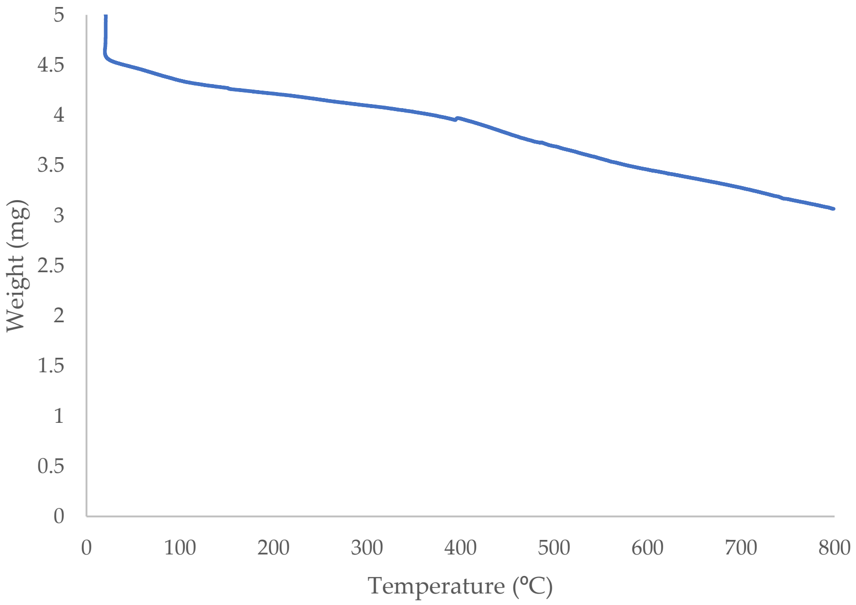

Figure S2. Degradation temperatures of a [Si][N_{3444}]Cl sample by Thermogravimetric analysis (TGA).

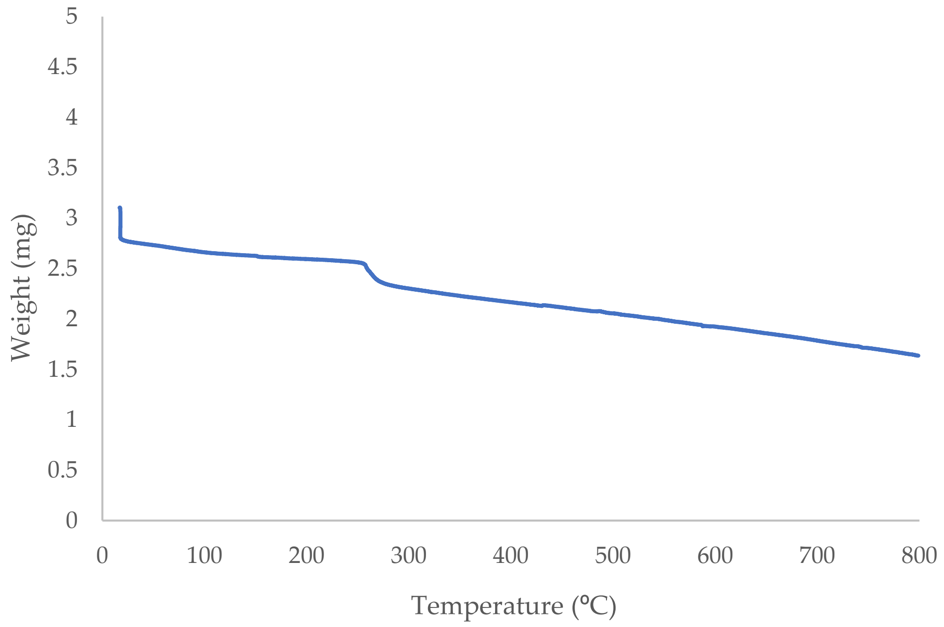

Figure S3. Degradation temperatures of a [Si][N_{3888}]Cl sample by Thermogravimetric analysis (TGA).

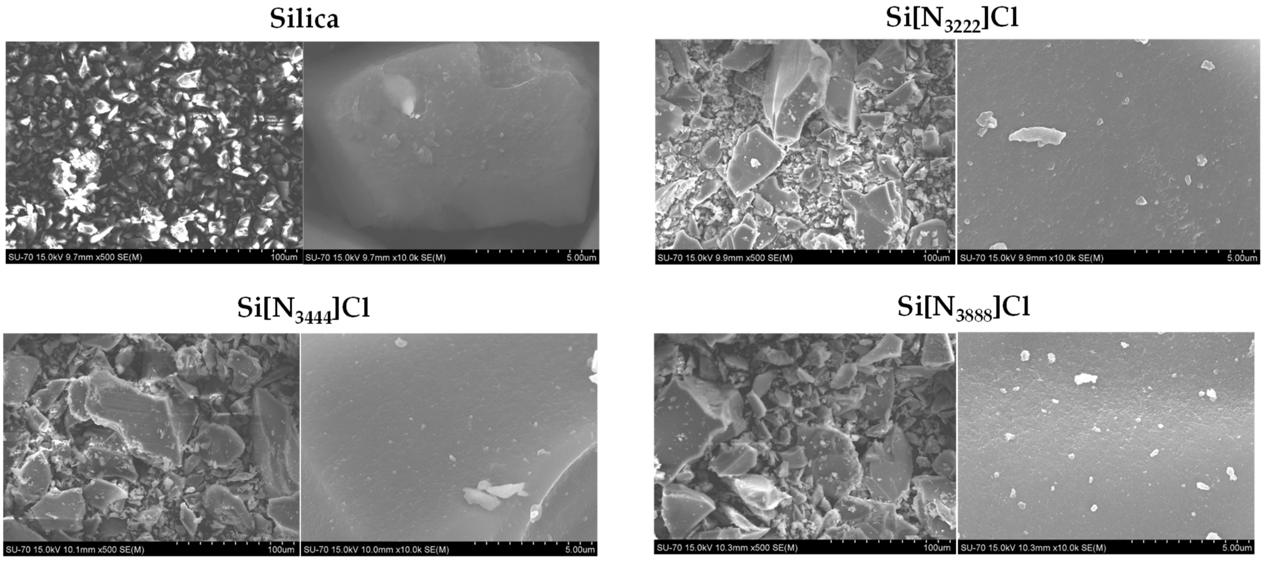

Figure S4. SEM images of silica and synthesized SILs (two different magnifications).

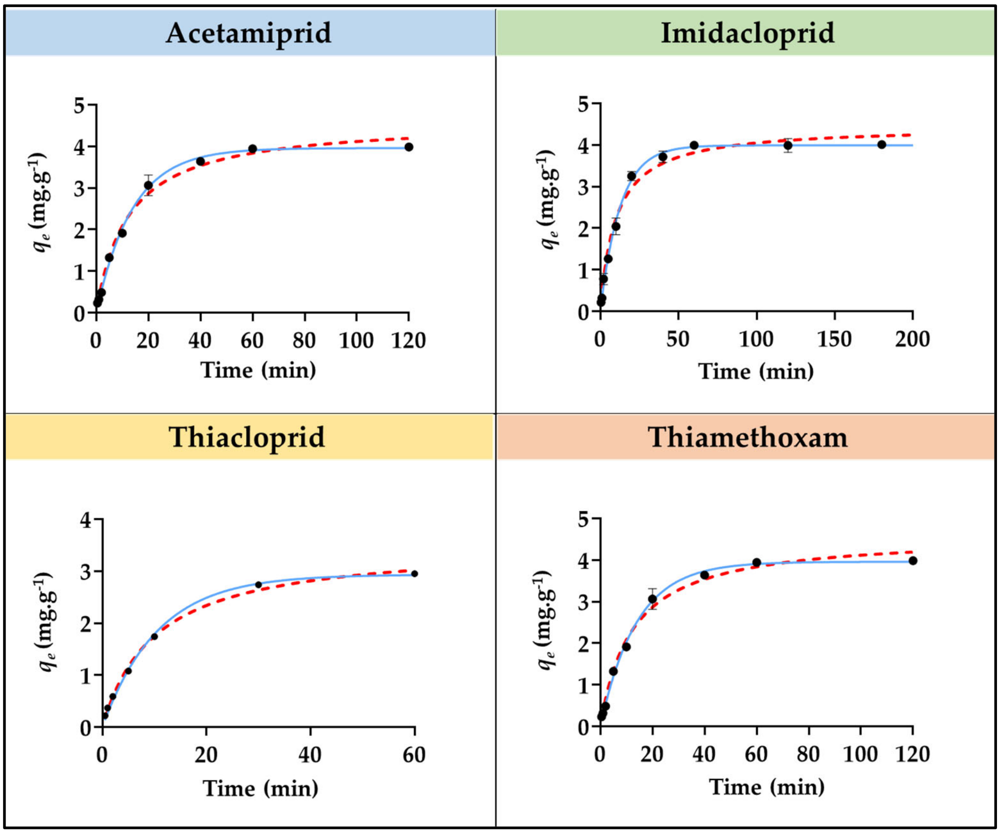

Figure S5. Adsorption kinetics curves for acetamiprid, imidacloprid, thiacloprid, and thiamethoxam insecticides with AC. The black dots correspond to the experimental data, the blue continuous line correspond to the adjustment of the experimental data with the Pseudo first-order model and the red dashed line corresponds to the adjustment of the experimental data with the Pseudo second-order model.

Table S1. Concentration of acetamiprid in the solid phase (q_e) for each contact time, with Si][N_{3222}]Cl, [Si][N_{3444}]Cl, [Si][N_{3888}]Cl, and the respective standard deviation (σ).

t (min)	Si[N_{3222}]Cl		Si[N_{3444}]Cl		Si[N_{3888}]Cl	
	q_e (mg g^{-1})	σ	q_e (mg g^{-1})	σ	q_e (mg g^{-1})	σ
0.5	0.962	9.00×10^{-9}	1.414	6.38×10^{-2}	0.842	1.686×10^{-2}
1	1.043	4.65×10^{-3}	---	---	1.245	6.719×10^{-2}
2	1.061	1.94×10^{-7}	1.686	6.09×10^{-3}	1.854	4.556×10^{-2}
5	1.085	6.55×10^{-3}	1.723	1.78×10^{-4}	1.970	2.425×10^{-2}
10	1.067	3.22×10^{-3}	1.739	1.50×10^{-2}	1.954	5.676×10^{-3}
20	1.007	2.50×10^{-3}	1.792	1.58×10^{-2}	1.911	1.381×10^{-2}
40	1.025	9.34×10^{-3}	1.753	4.50×10^{-2}	1.975	1.674×10^{-2}
60	1.042	9.72×10^{-4}	1.773	7.74×10^{-3}	1.950	4.310×10^{-2}

Table S2. Concentration of imidacloprid in the solid phase (q_e) for each contact time, with Si][N_{3222}]Cl, [Si][N_{3444}]Cl, [Si][N_{3888}]Cl, and the respective standard deviation (σ).

t (min)	Si[N_{3222}]Cl		Si[N_{3444}]Cl		Si[N_{3888}]Cl	
	q_e (mg g^{-1})	σ	q_e (mg g^{-1})	σ	q_e (mg g^{-1})	σ
0.5	0.808	2.621×10^{-2}	0.767	4.459×10^{-2}	1.970	6.270×10^{-4}
1	0.891	5.918×10^{-2}	0.834	4.804×10^{-2}	2.173	2.833×10^{-2}
2	0.902	4.632×10^{-2}	0.884	4.908×10^{-2}	2.310	1.131×10^{-1}
5	0.902	5.557×10^{-2}	1.127	4.427×10^{-2}	2.434	3.608×10^{-2}
10	0.884	1.517×10^{-2}	1.237	2.444×10^{-2}	2.479	3.849×10^{-2}
20	0.866	2.638×10^{-2}	1.270	1.290×10^{-2}	2.510	1.722×10^{-3}
40	0.833	2.526×10^{-2}	1.340	5.593×10^{-2}	2.424	2.618×10^{-2}
60	0.849	1.538×10^{-2}	1.398	3.339×10^{-3}	2.557	2.173×10^{-2}

Table S3. Concentration of thiacloprid in the solid phase (q_e) for each contact time, with Si][N_{3222}]Cl, [Si][N_{3444}]Cl, [Si][N_{3888}]Cl, and the respective standard deviation (σ).

t (min)	Si[N_{3222}]Cl		Si[N_{3444}]Cl		Si[N_{3888}]Cl	
	q_e (mg g^{-1})	σ	q_e (mg g^{-1})	σ	q_e (mg g^{-1})	σ
0.5	0.787	8.884×10^{-2}	0.532	1.501×10^{-1}	1.131	2.919×10^{-1}
1	1.362	7.250×10^{-3}	0.949	1.122×10^{-1}	1.856	5.769×10^{-2}
2	1.490	5.910×10^{-3}	1.148	3.674×10^{-2}	1.983	9.613×10^{-2}
5	1.613	5.153×10^{-2}	1.726	6.964×10^{-2}	2.241	6.359×10^{-2}
10	1.640	3.166×10^{-2}	1.067	5.527×10^{-2}	1.067	5.155×10^{-2}
30	1.663	1.126×10^{-2}	2.137	3.170×10^{-2}	2.232	2.475×10^{-2}
60	1.698	6.355×10^{-3}	2.192	4.507×10^{-2}	2.242	6.839×10^{-2}

Table S4. Concentration of thiamethoxam in the solid phase (q_e) for each contact time, with Si][N_{3222}]Cl, [Si][N_{3444}]Cl, [Si][N_{3888}]Cl, and the respective standard deviation (σ).

t (min)	Si[N_{3222}]Cl		Si[N_{3444}]Cl		Si[N_{3888}]Cl	
	q_e (mg g^{-1})	σ	q_e (mg g^{-1})	σ	q_e (mg g^{-1})	σ
0.5	0.812	2.364×10^{-2}	0.263	7.797×10^{-2}	1.091	1.142×10^{-1}
1	0.909	2.141×10^{-2}	0.339	3.810×10^{-2}	1.427	1.287×10^{-1}
2	0.947	1.305×10^{-2}	0.514	2.188×10^{-2}	1.844	1.619×10^{-2}
5	0.908	9.633×10^{-3}	0.572	5.763×10^{-2}	1.979	3.026×10^{-2}
10	1.067	2.062×10^{-2}	1.067	5.625×10^{-2}	1.067	7.247×10^{-2}
20	0.860	2.343×10^{-2}	0.672	2.685×10^{-2}	1.946	4.867×10^{-2}
40	0.814	1.976×10^{-3}	0.649	2.644×10^{-2}	1.930	9.226×10^{-2}
60	0.814	1.625×10^{-7}	0.697	2.570×10^{-2}	1.982	3.511×10^{-2}

Table S5. Concentration of acetamiprid, imidacloprid, thiacloprid, and thiamethoxam in the solid phase (q_e) for each contact time, with AC, and the respective standard deviation (σ).

t (min)	Acetamiprid		Imidacloprid		Thiacloprid		Thiamethoxam	
	q_e (mg g^{-1})	σ	q_e (mg g^{-1})	σ	q_e (mg g^{-1})	σ	q_e (mg g^{-1})	σ
0.5	0.235	1.76×10^{-2}	0.220	5.28×10^{-2}	0.222	6.43×10^{-3}	0.235	1.76×10^{-2}
1	0.322	3.64×10^{-3}	0.324	7.27×10^{-2}	0.373	1.27×10^{-3}	0.322	3.64×10^{-3}
2	0.491	3.47×10^{-2}	0.782	1.15×10^{-1}	0.592	2.60×10^{-3}	0.491	3.47×10^{-2}
5	1.33	6.96×10^{-2}	1.27	5.47×10^{-2}	1.08	2.57×10^{-3}	1.33	6.96×10^{-2}
10	1.07	3.00×10^{-3}	1.07	1.74×10^{-1}	1.07	0.00	1.07	3.00×10^{-3}
20	3.06	2.15×10^{-1}	3.25	9.29×10^{-2}	2.74	0.00	3.06	2.15×10^{-1}
40	3.64	1.42×10^{-2}	3.72	1.19×10^{-1}	2.95	1.27×10^{-3}	3.64	1.42×10^{-2}
60	3.94	3.42×10^{-2}	4.00	6.96×10^{-2}	---	---	3.94	3.42×10^{-2}
120	3.99	4.50×10^{-2}	3.99	1.44×10^{-1}	---	---	3.99	4.50×10^{-2}
180	---	---	4.01	5.62×10^{-2}	---	---	---	---

Table S6. Adsorption efficiencies at equilibrium and at 10 minutes of contact of imidacloprid, acetamiprid, thiacloprid and thiamethoxam with [Si][N_{3222}]Cl, [Si][N_{3444}]Cl, [Si][N_{3888}]Cl and AC.

Pesticide	Material	Adsorption efficiency (%)	
		At equilibrium	After 10 min.
Imidacloprid	[Si][N_{3222}]Cl	22.4	22.4
	[Si][N_{3444}]Cl	35.4	35.4
	[Si][N_{3888}]Cl	62.3	62.3
	AC	100.0	50.0
Acetamiprid	[Si][N_{3222}]Cl	28.5	28.5
	[Si][N_{3444}]Cl	47.5	47.5
	[Si][N_{3888}]Cl	50.4	50.4
	AC	98.9	48.8
Thiacloprid	[Si][N_{3222}]Cl	57.5	57.5
	[Si][N_{3444}]Cl	72.6	64.7
	[Si][N_{3888}]Cl	77.0	77.0
	AC	99.5	58.9
Thiamethoxam	[Si][N_{3222}]Cl	22.6	22.6
	[Si][N_{3444}]Cl	18.2	18.2
	[Si][N_{3888}]Cl	47.3	47.3
	AC	100.0	52.7

Table S7. Maximum equilibrium concentration (q_e) and aadsorption efficiencies (AE%) of [Si][C_3C_1im]Cl, [Si][N_{3114}]Cl [Si][N_{3222}]Cl, [Si][N_{3444}]Cl, [Si][N_{3888}]Cl for imidacloprid, obtained in the initial screening adsorption studies

Material	q_e (mg L^{-1})	AE (%)
**[Si][C_3C_1im]Cl**	0	0
**[Si][N_{3114}]Cl**	0.03	1.52
**[Si][N_{3222}]Cl**	0.45	23.9
**[Si][N_{3444}]Cl**	0.94	50.2
**[Si][N_{3888}]Cl**	1.08	57.4

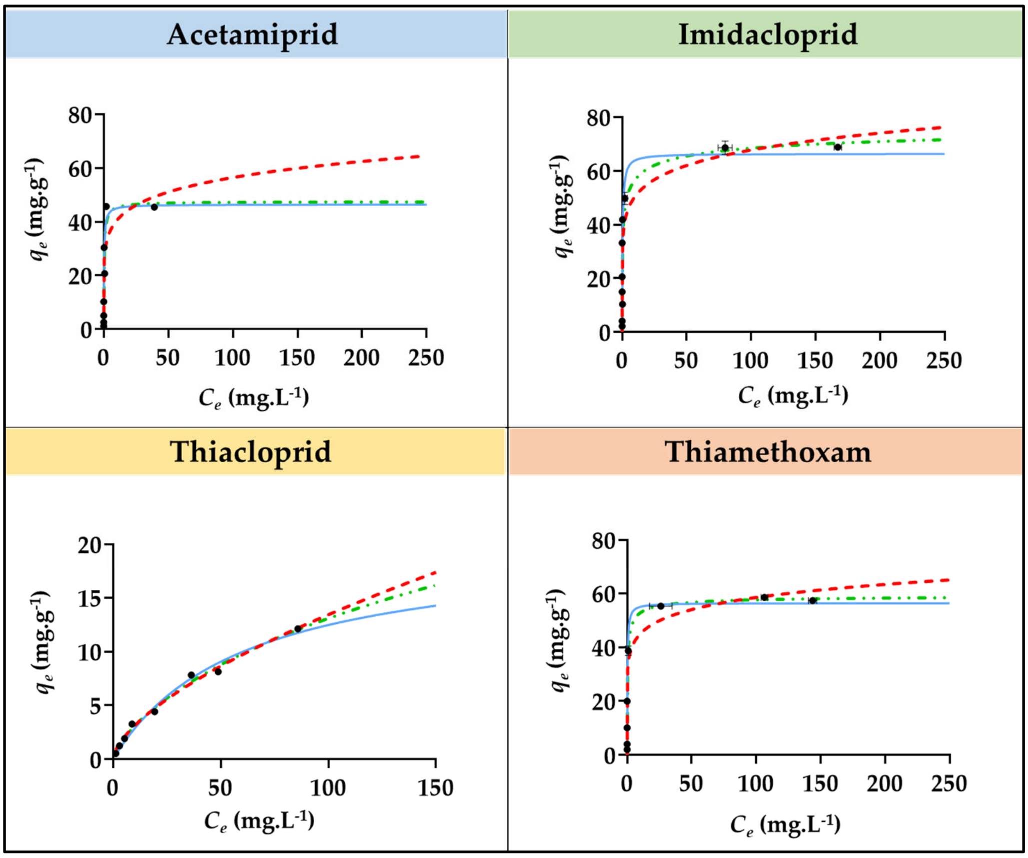

Figure S6. Adsorption isotherms curves for imidacloprid, acetamiprid, thiacloprid, and thiamethoxam insecticides with AC. The black dots correspond to experimental data, the continuous line correspond to an adjustment of the experimental data with the Langmuir model, the dashed line correspond to an adjustment of the experimental data with Freundlich model, and the dashed and pointed line correspond to adjustment of the experimental data with SIPS model.

Table S8. Equilibrium concentration of acetamiprid after adsorption (C_e), concentration of adsorbate in the solid phase (q_e), with respective standard deviations (σ), for [Si][C_{3222}]Cl and [Si][N_{3444}]Cl.

C_e (mg L^{-1})	Si[N3$_{2222}$]Cl		C_e (mg L^{-1})	Si[N_{3444}]Cl	
	q_e (mg g^{-1})	σ		q_e (mg g^{-1})	σ
208.8	13.383	3.617×10^{-1}	189.6	16.770	5.810×10^{-1}
162.5	12.295	3.074×10^{-1}	146.8	15.262	6.074×10^{-1}
116.7	8.678	0.000×10^{0}	105.2	10.945	3.351×10^{-1}
78.4	5.735	1.191×10^{-1}	68.8	7.384	1.898×10^{-1}
38.4	2.830	0.000×10^{0}	34.1	3.656	2.450×10^{-1}
18.4	1.493	9.417×10^{-3}	15.8	2.088	4.901×10^{-3}
9	0.814	0.000×10^{0}	7.7	1.033	1.861×10^{-3}
4	0.313	9.761×10^{-4}	3.3	0.465	1.937×10^{-3}

Table S9. Equilibrium concentration of acetamiprid after adsorption (C_e), concentration of adsorbate in the solid phase (q_e), with respective standard deviations (σ), for [Si][N_{3888}]Cl and AC.

C_e (mg L^{-1})	Si[N_{3888}]Cl		C_e (mg L^{-1})	AC	
	q_e (mg g^{-1})	σ		q_e (mg g^{-1})	σ
161	21.998	3.767×10^{-1}	39.2	45.453	2.803×10^{-2}
131	18.974	4.724×10^{-1}	2.000	45.680	1.008×10^{-1}
95.4	13.637	5.929×10^{-2}	0.300	30.333	9.027×10^{-5}
61.1	9.082	2.711×10^{-1}	0.700	20.681	0.000×10^{0}
27.6	4.928	4.841×10^{-2}	0.100	10.264	0.000×10^{0}
13.1	2.528	9.380×10^{-2}	0.00	5.010	0.000×10^{0}
7.60	1.118	0.000×10^{0}	0.00	2.584	0.000×10^{0}
2.80	0.559	0.000×10^{0}	0.00	1.144	0.000×10^{0}

Table S10. Equilibrium concentration of imidacloprid after adsorption (C_e), concentration of adsorbate in the solid phase (q_e), with respective standard deviations (σ), for [Si][C_{3222}]Cl, and [Si][N_{3444}]Cl.

C_e (mg L^{-1})	Si[N_{3222}]Cl		C_e (mg L^{-1})	Si[N_{3444}]Cl	
	q_e (mg g^{-1})	σ		q_e (mg g^{-1})	σ
195	13.069	1.455×10^{0}	190	13.743	1.991×10^{0}
154	9.838	1.265×10^{0}	154	10.317	1.132×10^{0}
113	6.346	1.793×10^{-1}	115	9.297	1.420×10^{0}
74.8	5.230	9.467×10^{-1}	74.4	5.168	3.898×10^{-1}
56.9	4.022	7.637×10^{-1}	58.0	3.851	4.546×10^{-1}
38.1	2.735	7.084×10^{-1}	37.7	2.864	1.874×10^{-1}
14.4	1.208	2.456×10^{-1}	14.8	1.136	1.575×10^{-1}
8.70	0.452	8.722×10^{-2}	9.30	0.338	3.707×10^{-1}
388	23.167	1.456×10^{0}	418	37.243	1.149×10^{-1}
468	27.353	2.348×10^{-1}	306	25.944	1.565×10^{-1}
314	22.588	1.679×10^{0}	---	---	---

Table S11. Equilibrium concentration of imidacloprid after adsorption (C_e), concentration of adsorbate in the solid phase (q_e), with respective standard deviations (σ), for [Si][N_{3888}]Cl and AC.

C_e (mg L^{-1})	Si[N_{3888}]Cl		C_e (mg L^{-1})	AC	
	q_e (mg g^{-1})	σ		q_e (mg g^{-1})	σ
134	25.642	8.172×10^{-1}	2.20	49.746	1.982×10^{0}
100	20.321	1.641×10^{-1}	0.400	41.858	8.364×10^{-1}
68.8	15.571	1.194×10^{-1}	0.100	33.138	6.886×10^{-1}
42.7	11.418	7.373×10^{-1}	0.00	20.546	1.910×10^{-1}
32.6	9.019	3.340×10^{-1}	0.00	14.979	4.452×10^{-1}
20.2	6.039	2.689×10^{-1}	0.300	10.368	1.627×10^{-1}
7.40	2.626	2.254×10^{-1}	0.00	4.127	1.004×10^{-1}
3.40	1.512	1.103×10^{-1}	0.00	2.194	6.606×10^{-2}
303	42.683	1.219×10^{0}	167	68.829	5.840×10^{-1}
357	48.671	4.910×10^{-1}	79.9	68.646	2.152×10^{0}
247	36.304	3.605×10^{1}	---	---	---

Table S12. Equilibrium concentration of thiacloprid after adsorption (C_e), concentration of adsorbate in the solid phase (q_e), with respective standard deviations (σ), for [Si][N_{3222}]Cl and [Si][N_{3444}]Cl.

C_e (mg L^{-1})	Si[N_{3222}]Cl		C_e (mg L^{-1})	Si[N_{3444}]Cl	
	q_e (mg g^{-1})	σ		q_e (mg g^{-1})	σ
101.0	15.866	4.809×10^{-1}	84.0	21.065	3.493×10^{-1}
64.6	10.399	8.495×10^{-2}	54.8	13.269	3.336×10^{-1}
49.0	8.702	1.627×10^{-1}	42.6	11.086	2.215×10^{-1}
28.1	5.888	2.127×10^{-1}	25.9	6.588	1.306×10^{-1}
15.4	3.123	2.069×10^{-1}	12.7	4.151	1.678×10^{-1}
9.50	2.344	2.356×10^{-1}	8.10	2.693	9.503×10^{-2}
6.40	1.092	6.426×10^{-2}	5.10	1.622	4.120×10^{-2}
3.10	0.550	8.286×10^{-3}	2.10	0.908	2.451×10^{-2}

Table S13. Equilibrium concentration of thiacloprid after adsorption (C_e), concentration of adsorbate in the solid phase (q_e), with respective standard deviations (σ), for [Si][N_{3888}]Cl and AC.

C_e (mg L^{-1})	Si[N_{3888}]Cl		C_e (mg L^{-1})	AC	
	q_e (mg g^{-1})	σ		q_e (mg g^{-1})	σ
80.7	23.178	5.367×10^{-1}	85.9	12.110	0.000×10^{0}
53.1	14.006	5.880×10^{-1}	48.9	8.130	0.000×10^{0}
41.1	12.036	4.271×10^{-1}	36.4	7.830	0.000×10^{0}
24.4	7.001	2.227×10^{-1}	19.4	4.420	0.000×10^{0}
12.3	4.190	1.804×10^{-1}	8.90	3.270	0.000×10^{0}
8.50	2.643	2.004×10^{-2}	5.40	1.920	0.000×10^{0}
4.70	1.748	4.500×10^{-2}	3.00	1.250	0.000×10^{0}
2.10	0.938	2.684×10^{-2}	1.30	0.540	0.000×10^{0}

Table S14. Equilibrium concentration of thiamethoxam after adsorption (C_e), concentration of adsorbate in the solid phase (q_e), with respective standard deviations (σ), for [Si][N$_{3222}$]Cl and [Si][N$_{3444}$]Cl.

C_e (mg L^{-1})	Si[N$_{3222}$]Cl		C_e (mg L^{-1})	Si[N$_{3444}$]Cl	
	q_e (mg g^{-1})	σ		q_e (mg g^{-1})	σ
433	14.781	0.000×10^{0}	176	4.846	1.601×10^{0}
343	11.248	5.544×10^{-1}	87.7	2.688	1.485×10^{-1}
250	10.642	1.060×10^{0}	44.3	1.315	2.004×10^{-1}
164	7.281	2.478×10^{-1}	17.7	0.699	7.125×10^{-2}
81.2	4.130	1.694×10^{-1}	644	33.762	5.914×10^{-1}
40.2	2.143	8.187×10^{-3}	479	27.774	1.342×10^{0}
16.2	0.990	1.634×10^{-2}	324	16.711	1.076×10^{0}
8.10	0.464	3.158×10^{-2}	---	---	---
713	21.028	3.894×10^{-1}	---	---	---
538	16.624	1.020×10^{-1}	---	---	---

Table S15. Equilibrium concentration of thiamethoxam after adsorption (C_e), concentration of adsorbate in the solid phase (q_e), with respective standard deviations (σ), for [Si][N$_{3888}$]Cl and AC.

C_e (mg L^{-1})	Si[N$_{3888}$]Cl		C_e (mg L^{-1})	AC	
	q_e (mg g^{-1})	σ		q_e (mg g^{-1})	σ
375	25.831	2.231×10^{0}	144	57.279	9.922×10^{-1}
288	25.480	6.821×10^{-1}	107	58.489	3.415×10^{-1}
210	20.281	2.110×10^{0}	26.2	55.221	1.007×10^{-1}
135	12.633	1.212×10^{0}	0.900	38.695	1.526×10^{0}
59.0	7.896	1.444×10^{-1}	0.100	19.955	5.485×10^{-1}
27.4	4.561	3.580×10^{-2}	0.00	10.119	2.213×10^{-1}
10.0	1.936	2.311×10^{-2}	0.00	4.036	8.137×10^{-2}
5.40	0.954	5.558×10^{-2}	0.00	2.063	3.341×10^{-2}
607	40.044	1.104×10^{0}	---	---	---
468	32.462	3.358×10^{-1}	---	---	---

Table S16. Maximum adsorption capacity for acetamiprid, imidacloprid, thiacloprid, and thiamethoxam with [Si][N_{3888}]Cl, obtained by columns saturation assays, and their respective standard deviations (σ).

**Insecticide**	**q_e (mg g^{-1})**	**σ**
Imidacloprid	50.653	1.303
Acetamiprid	34.289	1.145
Thiacloprid	49.266	1.206
Thiamethoxam	45.744	0.351
